# A Concise Review on Multi-Omics Data Integration for Terroir Analysis in *Vitis vinifera*

**DOI:** 10.3389/fpls.2017.01065

**Published:** 2017-06-20

**Authors:** Pastor Jullian Fabres, Cassandra Collins, Timothy R. Cavagnaro, Carlos M. Rodríguez López

**Affiliations:** ^1^Environmental Epigenetics and Genetics Group, Plant Research Centre, School of Agriculture, Food and Wine, University of Adelaide, Glen OsmondSA, Australia; ^2^The Waite Research Institute, The School of Agriculture, Food and Wine, The University of Adelaide, Glen OsmondSA, Australia

**Keywords:** multi-omics, environment, *Vitis vinifera*, data integration, epigenetics, transcriptomics, metabolomics

## Abstract

*Vitis vinifera* (grapevine) is one of the most important fruit crops, both for fresh consumption and wine and spirit production. The term *terroir* is frequently used in viticulture and the wine industry to relate wine sensory attributes to its geographic origin. Although, it can be cultivated in a wide range of environments, differences in growing conditions have a significant impact on fruit traits that ultimately affect wine quality. Understanding how fruit quality and yield are controlled at a molecular level in grapevine in response to environmental cues has been a major driver of research. Advances in the area of genomics, epigenomics, transcriptomics, proteomics and metabolomics, have significantly increased our knowledge on the abiotic regulation of yield and quality in many crop species, including *V. vinifera*. The integrated analysis of multiple ‘omics’ can give us the opportunity to better understand how plants modulate their response to different environments. However, ‘omics’ technologies provide a large amount of biological data and its interpretation is not always straightforward, especially when different ‘omic’ results are combined. Here we examine the current strategies used to integrate multi-omics, and how these have been used in *V. vinifera*. In addition, we also discuss the importance of including epigenomics data when integrating omics data as epigenetic mechanisms could play a major role as an intermediary between the environment and the genome.

## Introduction

**FIGURE 1:**
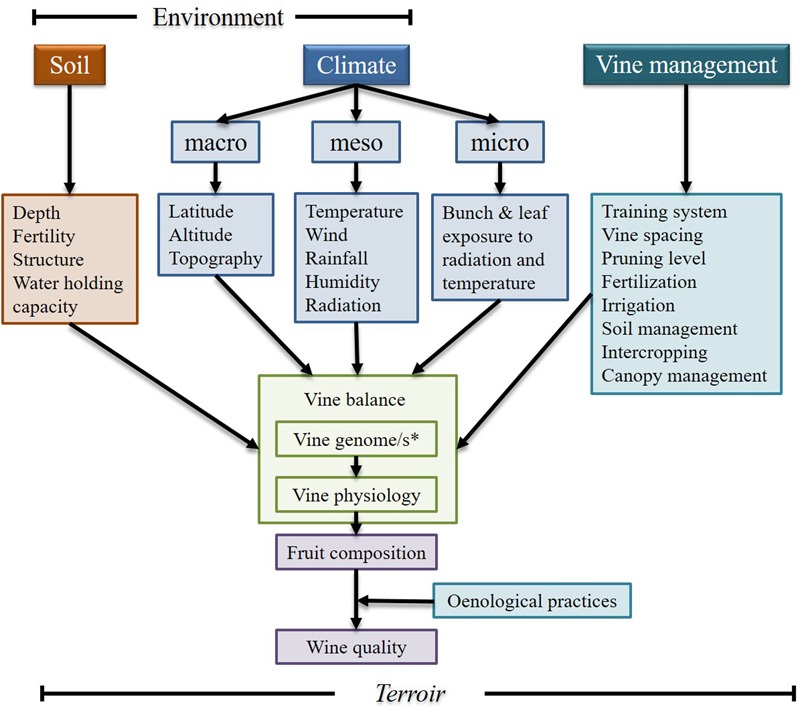
A conceptual view of some of the *terroir* factors that contribute to vine balance, fruit composition and wine quality. ^∗^Vine genome/s refers to the possibility of the scion and rootstock used in a vineyard could be from different varieties/species (Modified from: Smart et al., 1985 and reproduced with permission from the copyright holder).

Grapevine is one of the most economically important fruit crops, and it is largely used for wine production (FAO, 2012). Most the chemical compounds that give its unique characteristics to wine are synthesized during berry development (Conde et al., 2007). However, fruit/wine composition is strongly influenced by the interactions between the plant’s genome and the local growing conditions (including the vine management system), and the oenological practices of each winery (**Figure 1**), which could explain why it is so difficult to replicate a wine from a region outside that area.

*Terroir* is defined as the interactions between the plants, the environment and human factors (Gladstones, 2011) and it is frequently used to relate wine sensory attributes to its geographic origin (Van Leeuwen and Seguin, 2006). Although the relevance of *terroir* is still under debate (Anesi et al., 2015), a better understanding of how the environment affects grape berry composition can have a significant effect on viticulture. To achieve such an understanding, it is necessary to identify the elements that drive *terroir* and analyze the interaction between them and the grapevine.

## Decoding *Terroir*

*Terroir* has been long studied, through the characterization of the different environmental factors affecting berry composition and wine quality, and climate exerts the strongest effect on berry composition (Robinson et al., 2012). Soil physicochemical properties as well have been identified as an influential factor defining the uniqueness of berry composition by vines grown in a specific climate (Cheng et al., 2014; Zerihun et al., 2015). Grapevine microbiome community may play an important role determining wine quality (Burns et al., 2015; Bokulich et al., 2016). Efforts have been made to study the grapevine microbiome landscape in relation to the vegetative growth cycle of the plant (Pinto et al., 2014), the post-harvest treatment of berries (Salvetti et al., 2016) and provenance (Bokulich et al., 2016) (For a review on microbiome analysis see Ibrahim and Kumar, 2017). Less work has been done to elucidate the molecular mechanisms involved in the plant response to *terroir*. A strategy to better understand the genome and environment interaction is to use ‘omics’ technologies. Omics refers to high throughput technologies that generate a large amount of data for each sample, allowing a deeper insight of the mechanisms regulating biological systems.

## Analysis of *Terroir* Effect on Grape Composition Using Transcriptomics

Using transcriptomics is possible to study the grape’s complete set of RNA transcripts encoded by the genome using high throughput methods (Hale et al., 2005). Dal Santo et al. (2013) performed gene expression analysis in a single Corvina clone cultivated in 11 different vineyards for three consecutive years. Samples strongly clustered by season, known as a vintage effect, rather than common environmental conditions. However, the genes that showed more variation in expression between years were those involved in secondary metabolism, (mainly the biosynthesis of phenylpropanoids). Only when samples from a single vintage (i.e., 2008) were analyzed, it was observed that 5% of the studied annotated coding genes were differentially regulated under different growing conditions and agronomical practices. Anesi et al. (2015) complemented this study by analyzing the transcriptome and metabolome of the same cultivar. They identified metabolites that could describe a *terroir* signature for each vineyard. Moreover, it was possible to correlate terroir-sensitive metabolites with changes in the transcript level of genes involved in the biosynthesis of these metabolites. Similar results were obtained by Dal Santo et al. (2016) as they identified a clear correlation between gene expression and accumulation of phenylpropanoids and flavonoids in the variety Garganega grown at four different vineyards. Small RNA profiles have been analyzed to understand the interaction between genotype and environment in the varieties Sangiovese and Cabernet Sauvignon. *In silico* analysis suggests that microRNAs may be involved in berry development and the accumulation of secondary metabolites (Paim Pinto et al., 2016). Transcriptional analysis of berries from different regions has also shown that transcripts from the abscisic acid (ABA) biosynthesis pathway are among the most *terroir* sensitive genes (Sun et al., 2015). ABA is a plant hormone that regulates important steps in plant growth and development as well as play a key role in plant biotic and abiotic stress response (Cutler et al., 2010). ABA concentrations affect anthocyanin and flavonol accumulation (Koyama et al., 2010), suggesting a possible mechanism through which the environment affects grape berry composition and wine flavor and aroma.

## Analysis of *Terroir* Effect on Grape Composition Using Metabolomics

Metabolomics is defined as the identification and quantification of metabolites using high-throughput techniques (Cevallos-Cevallos et al., 2009). This technology can screen higher numbers of products than more traditional approaches (Pereira et al., 2006; Atanassov et al., 2009; Hong, 2011), while the use of non-targeted metabolomics approaches allows the identification of un characterized metabolites (Panighel et al., 2015). *Terroir* can be explored by analyzing berry metabolite composition through different analytical methods (For a review in grape and wine metabolomics see Cozzolino, 2016). Son et al. (2009) identified that differences in berry metabolomes associated to environmental regional differences (radiation and rainfall) could explain the observed differences in wine composition. Similar results were obtained by Tarr et al. (2013) who distinguished the metabolic signatures of different grapevine varieties. Metabolomic analysis has also been performed to identify chemical compounds that can be associated to regional wine quality traits (Gambetta et al., 2016, 2017). Roullier-Gall et al. (2014) assessed the metabolomics profiles of two different *terroirs*, which were just 2 km apart, over three vintages. Although vintage had the greatest effect in the berry’s metabolite composition, differences in fruit chemical composition associated to nearby *terroirs* could be detected when vintages were individually analyzed. This suggests that subtle geographical differences have a significant effect on grape/wine composition even when variability within vineyards can be relatively high (Mulas et al., 2011).

## Multi-Omics Integration

**FIGURE 2:**
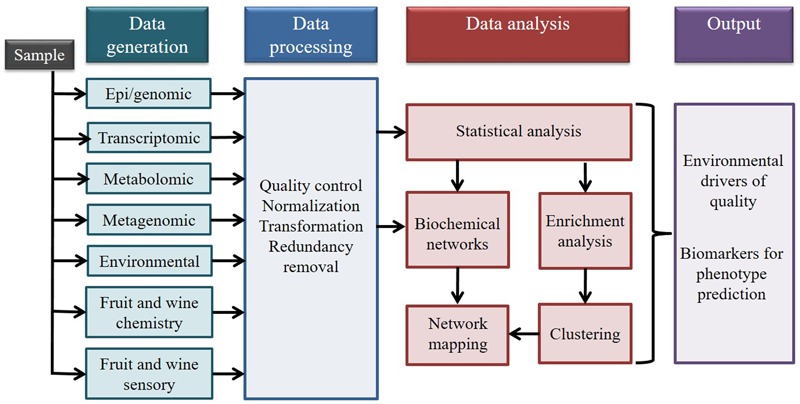
Data integration workflow for four omics technologies in addition to environmental data, and fruit and wine quality data (Modified from Wanichthanarak et al., 2015 and reproduced with permission from the copyright holder).

The aim of integrating multi-omic data is to reduce the gap between data generation and the ability to analyze and understand the biological mechanisms behind an organism’s response to environmental cues. The objective of multi-omic data integration is to combine different types of data to construct a model that can be used to predict complex traits and phenotypes (**Figure 2**). This approach also allows the identification of biomarkers and of previously unknown relationships between the datasets (Rajasundaram and Selbig, 2016). Through the integration of environmental information with genomic, epigenomic, transcriptomic, and metabolomic data, we hypothesize that it will be possible to better understand the effect of terroir at a molecular level. The use of a multi-omic approach will also help reduce the incidence of false positives generated from single source data sets (Aho, 2013; Ritchie et al., 2015). However, integration of multi-omics data is not a trivial task, because the diversity of characteristics of the data generated from the different high throughput technologies (machine sensitivity, error rate, data structure) makes its combination challenging.

## Approaches to Datasets Integration

Analysis of large data sets from different origins has been done using two main approaches: network models (NMs) and pathway analysis (PA). Both share the basic idea of storing the data in a clear and meaningful way. NMs use concepts from mathematical graph theory, to represent biological components (e.g., genes) as nodes and their interactions (physical, genetic or functional) as their links (For a review on NM applied to plant biology see Fukushima et al., 2014). NMs are classified as homogeneous or heterogeneous depending on the number of different levels of information integrated (Gligorijević and Pržulj, 2015). Homogenous approaches integrate datasets with the same type of nodes and therefore cannot analyze the connectivity between multiple datasets simultaneously. However, complex biological questions such as the molecular regulation of fruit composition in grapevine are increasingly being addressed through the integration of multiple layers of cellular information (Wong and Matus, 2017), including but not restricted to genomics, transcriptomics, proteomics and metabolomics, using heterogeneous methods. Bayesian networks (BNs) and Kernel-based methods (KBMs) are heterogeneous approaches commonly used for data integration (Zhang, 2009; Gligorijević and Pržulj, 2015). BNs are efficient detecting relationships hidden in noisy datasets but they are computationally demanding (Gligorijević and Pržulj, 2015) and are therefore, better suited for the interrogation of small datasets in hypothesis driven questions (Gligorijević and Pržulj, 2015) (i.e., the analysis of terroir on defined pathways such as those leading to the biosynthesis of metabolites related to fruit quality). KBMs are not as computationally demanding and so can integrate large molecular, structural and phenotypic datasets (Mizrachi et al., 2017), making them ideal for data driven terroir exploratory studies, biomarker discovery or for the reclassification of previously identified drivers of quality (Qi et al., 2008).

On the other hand, pathway analysis requires well documented biochemical pathways where omics data is combined to seek overrepresented groups (Wanichthanarak et al., 2015). For example, multiple co-inertia analysis (MCIA) can detect explanatory omic features even when they are not present in all datasets (Meng et al., 2014), which makes it attractive for the integration of terroir data from different studies. Random Forest implemented for pathway analysis (Pang et al., 2006), can be used to predict fruit/wine quality traits associated to terroir integrating multi-omic and phenotypic data as shown recently for potato (Acharjee et al., 2016).

Most of these multi-omics analysis approaches are pipelines that perform task sequences which share statistical methods (Bersanelli et al., 2016). Correlation analyses are the most common approaches performed to find relationships between the omics data. Simple correlation analyses, like Pearson or Spearman correlation, are widely used for multi-omics data integration (Rajasundaram et al., 2014; Rajasundaram and Selbig, 2016). Partial least square/projections to latent structures (PLS) and its extension, orthogonal partial least square (OPLS) (Tobias, 1995) have also been used for data integration from multi-omics results. Even though their predictive power is similar, OPLS results are much easier to interpret and outliers are quickly detected. OPLS can be used as a discriminate analysis (OPLS-DA), to identify differences between the overall data properties while removing systematic variation (Kirwan et al., 2012). However, these methods provided little insight when they are used in complex biological systems (highly multicollinear systems) (Wanichthanarak et al., 2015).

Modifications to these methods have been implemented to facilitate the interpretation of the data, for example, sparse PLS (sPLS) (Chun and Keleş, 2010) can better predict phenotypes through multi-omics data integration than previous methods (Rajasundaram et al., 2014). Orthogonal 2PLS (O2PLS), capable of dealing with unrelated systematic variation between datasets (Bouhaddani et al., 2016), has been successfully used for data integration of transcriptomics and metabolomics results from aspen under different light treatments (Bylesjö et al., 2007). Srivastava et al. (2013) used orthogonal projections to latent structures (OnPLS), an extension of O2PLS, to integrate transcriptomics, proteomics, and metabolomics data to construct a model that could identify biological relevant events in the oxidative stress response in poplar.

## Data Integration in *V. vinifera*

In plant science, most of data integration of omics results comes from model plants; however, there is an increase in publications on multi-omics data integration in *V. vinifera*. One of the first publications in multi-omics data integration in *V. vinifera* was the work of Zamboni et al. (2010). Integrating transcriptome, proteome and metabolome data, they identified stage specific biomarkers for berry development. Data integration was performed using two strategies, one hypothesis driven (i.e., a hypothesis was tested) and the other hypothesis free (i.e., discovery driven), in both cases principal component analysis (PCA), O2PLS and O2PLS-DA were used.

Using five different omics technologies and correlation analysis (PCA and Pearson correlation) together with biochemical pathway analysis (KEGG, PlantCyC and VitisCyC), Ghan et al. (2015) could differentiate biochemical characteristics from five different cultivars. Moreover, Anesi et al. (2015) studied the *terroir* effect in *V. vinifera* cultivar Corvina in seven different sites over a 3 years period using metabolome and transcriptome data. Using correlation analyses (PCA, PLS-DA and O2PLS-DA) they could identify a *terroir* signature in the berry metabolome composition for each growing site. Network analyses have been recently adopted to integrate grapevine multi omics results (Wong and Matus, 2017). For example, Palumbo et al. (2014) using network-based methods, identified “fight-club” nodes (genes with negatively correlated profiles) that may be relevant for the control of berry transition between development and ripening.

There are also online resources available that can help analyze omic data from *V. vinifera*. For example, VitisNet (Grimplet et al., 2009, 2012) offers manually annotated molecular networks (16,000 genes and 247 networks) where omics data can be loaded to visualize changes in the transcriptome, proteome and metabolome for a given experiment. VTCdb (Wong et al., 2013) is a gene co-expression database for *V. vinifera* that allows exploring transcription regulation. With more than 29,000 genes (95% of the predicted grapevine transcriptome) to query co-expression networks, VTCdb offers the possibility to analyze the transcriptional network of grapevine development, metabolism and stress response. VitisCyc (Naithani et al., 2014) is a grapevine metabolic pathway database that also allows omics data to be uploaded (transcriptome, proteome and metabolome) and to analyze changes in metabolic networks in each experiment. VESPUCCI (Moretto et al., 2016) is a manually annotated gene expression compendium exploratory tool that can be used to investigate grapevine’s gene expression patterns.

## Phenotypic Plasticity Through Epigenetic Modifications

Epigenetics is the study of heritable phenotypes that occur through modifications that alter DNA activity without modifying its basic nucleotide structure (Feil and Fraga, 2012). Many epigenetic mechanisms, acting in an interactive and redundant fashion (Grant-Downton and Dickinson, 2005; Berger et al., 2009), have been described to date, with DNA methylation probably being the best-studied of all (Rapp and Wendel, 2005). DNA methylation affects chromatin condensation in a rapid and reversible manner (Grativol et al., 2012). In turn, the regional level of chromatin condensation affects the transcriptional state of nearby genomic features such as genes and transposable elements (Zhang et al., 2006). Global changes in DNA methylation associated to local environments can be analyze using a myriad of methods (Kurdyukov and Bullock, 2016). Bisulfite modification of genomic DNA combined with whole genome sequencing (BS-Seq) is the gold standard for methylation analysis because it can assess an entire methylome with single base resolution (Krueger et al., 2012). However, due to their lower cost, other approaches such as next generation sequencing following the capture of the methylated fraction of the genome or its fragmentation using methylation sensitive restriction enzymes (Bock et al., 2010; Li et al., 2010; Kitimu et al., 2015) are better suited to study large number of samples. Both generate quantitative and qualitative information of the methylation status of a reduced but significant representation of the total genome.

Environmental signals are one of the elements that can have a major effect in modifying the DNA methylation patterns leading to gene expression changes that ultimately affect the plant phenotype (Feil and Fraga, 2012). The idea that the environment could modify the epigenetic status, and these modifications passed to the offspring (Tricker et al., 2013) or maintained as epigenetic memory on long lived organisms (Latzel et al., 2016), has attracted attention from scientists studying mechanisms involved in adaptation to local environments (Consuegra and Rodríguez López, 2016) and how these could be used to enhance crop performance (Rodríguez López and Wilkinson, 2015). There are many reported examples of how the environment affects the epigenome in natural environments and how epigenetic variations in plant populations could help to overcome the lack of genetic diversity (Fonseca Lira-Medeiros et al., 2010; Verhoeven et al., 2010).

One of the most well-known examples in which the environment affects the phenotype through epigenetic modifications is vernalization (Feil and Fraga, 2012). Through this process, plants in temperate regions mitigate the deleterious effects of low winter temperatures on flower and fruit development by breaking dormancy only after having been exposed to a cold period (Kumar et al., 2016). Unusual environmental conditions during dormancy such as high winter temperatures have been shown to exert a negative effect on fruit quality and yield on perennial crops requiring a vernalization period (Sugiura et al., 2012). Recent work in apple shows how methylation and expression levels of key genes involved in flowering and fruit set are modified by the level of chill received during bud dormancy (Kumar et al., 2016), indicating that the environmentally induced changes observed in fruit quality could be regulated by DNA methylation.

Together these studies suggest that the environment can have a long lasting phenotypic effect in plants through epigenetic changes without the need for genetic variation, and that epigenetic mechanisms could be working as intermediaries between environmental variation and the plant genome, and in this way, potentially contributing to plant phenotypic plasticity. Moreover, this mechanism could give plant populations a way of adapting to the local growing conditions (Platt et al., 2015; González et al., 2016). However, to our knowledge, almost all epigenetic studies done in *V. vinifera* have focused on the identification of commercial clones (Imazio et al., 2002; Schellenbaum et al., 2008; Ocaña et al., 2013) and on the assessment of *in vitro* culture on the epigenome (Baránek et al., 2015), there is, therefore, a lack of information of how the environment affects a grapevine’s epigenome and to what extent this interaction affects fruit quality. Until now, there are no studies looking at the epigenome to understand the control of gene expression in *V. vinifera* and how environmental signals can change the regulation of metabolic pathways through epigenetic modifications. In our view, the inclusion of epigenomic data on the analysis of the *terroir* effect will not only increase the resolution of analysis but will also help us to understand the regulatory mechanisms behind the observed differences.

## Conclusion

There is no doubt that the elements affecting grapevine growth and fruit composition are complex and multifarious. While the concept of *terroir* is widely discussed, the underlying mechanisms remain somewhat enigmatic. However, with the recent parallel development of omics technologies and of statistical approaches for their integration, we are reaching a point where it may be possible to overcome this challenge. The geographic delimitation of a terroir is the first challenge to overcome before its molecular characterization. This delimitation could be achieved 1. Empirically, based in the number of significantly different environmental subregions present in the study or/and 2. based on the traditionally defined wine regions. Moreover, the masking effect that environmental inter-annual variations can have over single year measurements demands the incorporation of data from multiple seasons to be able to determine terroirs with enough confidence. Ideally such seasons should be, from a weather perspective, variable within the range characteristic for the region of study to be able to capture its “normal terroir.”

Understanding how the genome, environment and viticulture practices interact to affect fruit quality will allow us the opportunity to implement agricultural practices aimed to obtain the desired fruit characteristics for every climate/cultivar combination (Jones and Davis, 2000), leading to more efficient use of resources and better management of vineyards. In addition, grape growers can maximize the *terroir* effect on the grapevine to highlight the uniqueness of their vineyards ultimately increasing their industrial competitiveness. We propose that the integration of multi-omic and environmental datasets will contribute to a better understanding of the drivers of the *terroir* effect in grapevine. Moreover, multiple dataset data integration will increase our understanding of the molecular mechanisms involved in the regulation of multifactorial genome by environment interactions. Finally, it is increasingly recognized that plants are involved in complex interactions their soil and epiphytic microbiomes, which can affect their phenotype (Mueller and Sachs, 2015). The ‘omics’ era gives us the ability to explore the nature and consequences of biotic/abiotic interactions and so, a future challenge will be to bring the concept of the holobiont (the plant host plus its microbiomes) into the analysis of terroir and its effect on grapevine growth and fruit composition.

## Author Contributions

PF, TC, CC, and CR designed and wrote the manuscript.

## Conflict of Interest Statement

The authors declare that the research was conducted in the absence of any commercial or financial relationships that could be construed as a potential conflict of interest.
